# Galangin reduces MPTP-induced dopamine neuron injury via the autophagy dependent-PI3K/AKT pathway

**DOI:** 10.3389/fnagi.2025.1568002

**Published:** 2025-04-04

**Authors:** Liping Huang, Qiaofeng Li, Jingyi Wu, Yingying He, Junwei Huang, Sipeng Xie, Canfeng Yang, Qingling Ruan, Zhongliu Zhou, Minzhen Deng

**Affiliations:** ^1^School of Chemistry and Chemical Engineering, Western Guangdong Characteristic Biomedical Engineering Technology Research Center, Lingnan Normal University, Zhanjiang, China; ^2^Mangrove Institute, Lingnan Normal University, Zhanjiang, China; ^3^State Key Laboratory of Traditional Chinese Medicine Syndrome/ Department of Neurology, The Second Affiliated Hospital of Guangzhou University of Chinese Medicine, Guangzhou, China; ^4^Guangdong Provincial Key Laboratory of Research on Emergency in TCM, Guangzhou, China

**Keywords:** Galangin, Parkinson’s disease, autophagy, network analysis, transcriptomics

## Abstract

**Introduction:**

Research has confirmed that Galangin can attenuate autophagy and protect dopaminergic neurons. This study aims to clarify whether Galangin attenuates dopaminergic neuron injury by regulating the PI3K/AKT pathway in Parkinson’s disease (PD) model mice.

**Methods:**

The study explores the mitigating effects of Galangin on PD processes by administering 1-methyl-4-phenyl-1,2,3,6-tetrahydropyridine (MPTP) to induce the condition. Techniques including network analysis, transcriptomic analysis, rotarod test, enzyme-linked immunosorbent assay (ELISA), qRT-PCR, western blotting, immunohistochemistry, immunofluorescence, and hematoxylin–eosin (HE) were employed to unveil the molecular changes induced by Galangin.

**Results:**

The network pharmacological analysis showed 301 targets related to Galangin, and 2,858 genes related to PD. Galangin treatment can improve the motor coordination of PD model mice, reduce damage to neurons in the brain, improve the antioxidant capacity and reduce the inflammatory damage of brain tissue. Additionally, Galangin suppressed mRNA expression of PD markers (IL-1β, TNF-*α*, IL-6, SRC and PTGS2), elevated protein levels of GSH-Px, SOD, P-PI3K, P-CREB, P-AKT, TH, BDNF and P62, while decreasing α-syn, SRC, MDA, Beclin-1 and LC3B expression. Moreover, the expression of significantly different genes in the Galangin-treated group and model group analyzed by transcriptomics was basically consistent with the qRT-PCR verification results.

**Conclusion:**

Galangin supresses Beclin-1-dependent autophagy and upregulates the PI3K/AKT signaling pathway to attenuate the neuroinflammatory injury and improve motor coordination ability in PD mice induced by MPTP.

## 1 Introduction

With the aging of the population, the incidence and prevalence of Parkinson’s disease (PD) continue to increase, which brings a heavy burden to society and the family, and has been widely concerned in the medical and genetic circles (Nair et al., 2018). The pathological mechanism of PD is related to the loss of dopamine (DA) neurons and *α*-synuclein (α-syn) aggregation in the substantia nigra dopaminergic neurons, oxidative stress, neuroinflammatory effects, mitochondrial dysfunction, endoplasmic reticulum stress disorder, neurotoxicity, uncontrolled autophagy, kinase signaling pathway, neurotrophic factor loss and nerve cell apoptosis (Ping and Geng, 2023; Nagoshi, 2018). The pathogenesis of PD is still unclear, and there is currently no known cure for PD, and the primary goal of treatment is to improve and slow the progression of clinical symptoms (Bhidayasiri et al., 2023). Therefore, there is a pressing necessity to find an effective treatment that alleviates the death of dopaminergic neurons in the midbrain and protects brain function for the treatment of PD.

At present, the western drugs against PD mainly included DA receptor agonists, monoamine oxidase B inhibitors, cholinergic inhibitors, catechol-O-methylase inhibitors (Laurence, 2020). Western medicine works well for treating PD and improves its motor symptoms. However, it has a poor curative effect on the non-motor symptoms of PD and is prone to major side effects like efficacy attenuation, dose end phenomenon, and transaction disorder. In recent years, metabolites derived from Chinese medicine have received increasing attention as a result of their clear neuroprotective effects. *Alpinia officinarum* Hance is a perennial herb belonging to the *Alpinia* genus of the ginger family. Its rhizome is a commonly used medicinal herb and spice in Asian and European countries, and it is distributed in tropical regions such as the Leizhou Peninsula in Guangdong, Hainan, and southern Guangxi in China. Galangin is a naturally occurring flavonoid (3, 5, 7-trihydroxyflavonoid) extracted from the rhizome of *Alpinia officinarum* Hance that shows a variety of biological activities, including anti-oxidant and anti-inflammatory functions (Aladaileh et al., 2019; Huang et al., 2017). Additionally, it is also found in abundance in propolis and plantain. At present, there are few studies on the treatment of PD by Galangin. In the brains of PD model rats, Galangin has been shown in two studies to have a protective impact on dopaminergic neurons (Chen et al., 2017; Chen Q. et al., 2022), reflecting that Galangin has a significant anti-PD effect, but its mechanism still needs to be further explored. In addition, studies have confirmed that Galangin can regulate oxidative stress, inflammation, and apoptosis in diabetic rats to alleviate diabetic cardiomyopathy (Abukhalil et al., 2021). Our previous study has demonstrated that treatment with Galangin regulated autophagy and effectively reduced p-tau, A*β*_42_ and β-secretase levels in AD model cells (Huang et al., 2019). Natural metabolites can thoroughly reflect the multiple pathways and targets, while personalized treatment, as much as possible reduces adverse reactions, and improve the prognosis and the life quality of PD patients. Additionally, further investigation is necessary to comprehensively elucidate the underlying mechanism through which Galangin exerts its anti-PD properties. Therefore, based on network analysis and transcriptomic analysis and molecular docking techniques to predict the pathways and targets of Galangin that are involved in PD therapy.

The PI3K/AKT signaling pathway plays a crucial role in the regulation of cellular proliferation, apoptosis and metabolism. The abnormally expressed PI3K/AKT signaling pathway is closely related to the occurrence and development of various neurological diseases. Therefore, this pathway has been extensively studied and is a potential target for disease treatment (Chen K. et al., 2022; Chen et al., 2023; Yang et al., 2023). The PI3K enzyme is an intracellular phosphatidyl inositol kinase that has the activity of serine/threonine kinase and phosphatidyl inositol kinase. AKT is a serine/threonine kinase that plays a crucial role in regulating various cellular processes including cell growth, survival, transcription, and protein synthesis and acts as a central component of the PI3K/AKT signaling pathway. PIP3 produced by PI3K can further activate AKT by activating phosphoinositol-dependent protein kinase 1 (PDK1) (Guo et al., 2017). Activated AKT activates downstream transcription factors and regulates transcription of related target genes. The PI3K/AKT/mTOR signaling pathway is an important pathway that negatively regulates autophagy, which is widely existing in cells. PI3K inhibits autophagy by activating AKT. The downstream target gene AKT can suppress autophagy by modulating the expression of FOXO and inducing transcription factor activating protein-1 (AP-1), thereby inhibiting the expression of autophagy-related genes (Calnan and Brunet, 2008; Eijkelenboom and Burgering, 2013). Autophagy is a double-edged sword, insufficient autophagy can lead to apoptosis under adverse environmental conditions, however, continuously activated autophagy can also lead to cell death (Koch et al., 2020; He et al., 2014). In addition, studies have shown that the PI3K/AKT signaling pathway can inhibit the activation of downstream mediator nuclear factor-κB (NF-κB), thereby reducing the release of kidney inflammatory indicators of tumor necrosis factor-*α* (TNF-α) and interleukin-6 (IL-6) (Hong et al., 2017). In brief, autophagy plays a dual role in cell survival; weakened autophagy or excessive autophagy is detrimental to cell survival. However, how Galangin properly regulates the PI3K/AKT signaling pathway and autophagy to enhance motor coordination remains elusive. Thus, in the present study, we aimed to investigate the effects of Galangin on motor coordination and autophagy alleviation in MPTP-induced mice. Additionally, for the first time, we revealed the mechanisms of Galangin in alleviating the injury of dopaminergic neurons by network analysis, molecular docking and transcriptomic analysis. Based on network analysis, molecular docking and transcriptomic analysis, we hypothesize that Galangin can alleviate neuroinflammation and inhibit Beclin-1 dependent autophagy by mediating the PI3K/AKT signaling pathway.

## 2 Materials and methods

### 2.1 Identifying targets of Galangin

We searched and confirmed the potential targets of the Galangin monomer through TCMSP database,^1^ Swiss Target Prediction,^2^ PharmMapper^3^ (Peng et al., 2020; Zhang et al., 2021). The obtained target names were converted into corresponding gene names through UniProt database,^4^ and the final target proteins related to Galangin were obtained by combining and deduplicating.

### 2.2 Identifying PD-related targets in Galangin

Using “Parkinson’s disease” as the keyword, disease targets were retrieved from Genecards,^5^ DisGeNET,^6^ and OMIM,^7^ with a filtering criterion in GeneCards of a score value higher than the median. The intersection targets of Galangin against Parkinson’s disease were obtained by overlapping the component targets with the disease targets. The common target genes screened from the Genecards, DisGeNET and OMIM databases and the targets of Galangin are consistent with this article (Shailima et al., 2021). VENNY2.1^8^ was adopted to establish Venn diagrams and obtain the common targets.

### 2.3 Protein–protein interaction (PPI) network construction

To further study the interaction targets between Galangin and PD, STRING,^9^ was used to identify known and predict interactions between proteins (Szklarczyk et al., 2019). Then, we removed the free proteins, and the correlation data between the targets were exported and constructed a network by the Cytoscape 3.9.1.

### 2.4 KEGG and GO enrichment analysis

In this study, the GO functions of enriched target genes were analyzed by the DAVID^10^ database (Dennis et al., 2003). We entered the UniProt ID of the protein, and the source of species was set as “*Homo sapiens*” to analyze the enriched biological processes, molecular functions, cellular components, and pathways related to the key proteins. The KEGG and GO enrichment were analyzed in a bioinformatics online tool.^11^

### 2.5 Animal experiment

Sixty specific pathogen free (SPF) grade National Institute of Health (NIH) male mice weighing 18–22 g were used in this study. The mice were provided by the Guangdong Medical Experimental Animal Center (Guangdong, China; certificate no.: SYXK (Guangdong) 2018-0002). The mice were raised in the Experimental Animal Center of Guangdong Traditional Chinese Medicine Hospital. All animal experiments were conducted in strict accordance with the Ethics Committee Guidelines of Guangdong Provincial Hospital of Traditional Chinese Medicine (No.2022039, April 25, 2022).

For observation of the therapeutic effect of Galangin on PD mice, sixty mice were randomly divided into the five groups (*n* = 10): control group, PD model group (MPTP, 30 mg/kg), madopar group (madopar + MPTP, 125 + 30 mg/kg), low-dose Galangin group (L-Galangin + MPTP, 25 + 30 mg/kg), medium-dose Galangin group (M-Galangin + MPTP, 50 + 30 mg/kg) and high-dose Galangin group (H-Galangin + MPTP, 100 + 30 mg/kg). The PD model mice was established as reported previously (Zhang et al., 2018) and the group administered with madopar serves as the positive control group in the experiment. The PD model mice were established by intraperitoneal injection of MPTP (30 mg/kg) once a day for 7 days. Mice were sacrificed after the behavioral tests by cervical vertebra dislocation, and then heart perfusion was performed by using saline. The hippocampus, striatum and mesencephalon were rapidly dissected out, frozen, and stored at −80°C for detection. Galangin (A0437, Chengdu must Bio-Technology CO., Ltd., purity = 99.94%) was prepared in 0.9% physiological saline and intragastric administration once daily for 28 days, meanwhile, the control group and the PD model group were given the same volume of saline.

After the administration was completed and behavioral tests were finished, the mice were anesthetized with 40 mg/kg sodium pentobarbital for euthanasia. The whole brain was then carefully removed on an ice plate. Half of the brains from each group (*n* = 6) were fixed in 4% paraformaldehyde for 24 h, followed by dehydration with a sucrose solution, embedding, and sectioning into 20 μm coronal slices for immunohistochemistry, immunofluorescence, HE and Nissl staining observations. Subsequently, the cerebral cortex, striatum, hippocampus, and midbrain tissues from each mouse were rapidly extracted and frozen in liquid nitrogen. The cerebral cortex from 8 mice per group was used for enzyme-linked immunosorbent assay, and the midbrain tissues from 6 mice per group were used for western blotting and quantitative polymerase chain reaction analyses.

### 2.6 Behavioral tests

#### 2.6.1 Rotarod test

A rotarod treadmill (ZB-200, Chengdu Thai union technology co., LTD, China) was used to evaluate motor coordination. Mice were placed on a slowly accelerating rod to keep balance and resist fatigue. Then we recorded the duration that a mouse kept standing or walking on the rotating rotarod at the speed of 30 r/min. A period of 300 s was taken as the maximum time of mice staying on the rotarod.

#### 2.6.2 Autonomous activity

Test for the mouse autonomous activity test, after the final dose administration, the mice from each group were placed in the mouse voluntary activity testing apparatus to acclimate for 5 min. Following the acclimation period, the number of movements made by the mice within a 5-min interval was recorded. Throughout the experiment, the surrounding environment was maintained quiet, with no movement of personnel, to prevent external factors from disturbing the mice.

#### 2.6.3 Pole climbing behavior

Pole climbing experiment select a wooden rod that is 50 cm long and 1.5 cm in diameter. Wrap the entire rod with tape to prevent the mice from slipping during the climbing process. During the acclimatization period, guide the mice to climb from the top to the bottom of the rod daily. After administration, train once a week. The measurement method is as follows: holding the tail of the mouse, place the mouse head-down at the top of the rod (with all four limbs on the top), and let it climb down naturally. Use a stopwatch to record the time it takes for the mouse to stand at the top of the rod as time A, and the time it takes for the mouse to climb to the bottom of the rod (with both front limbs touching the bottom platform) as time B. The total time the mouse spends climbing the rod is then calculated as time C, where C = A-B. Each mouse repeats the climbing test three times, with at least a 30-min interval between each attempt, and the average time is taken. If the mouse slips off or jumps out of the rod, the time for that attempt is not recorded, and the test is retaken after at least a 30-min interval.

### 2.7 Enzyme-linked immunosorbent assay (ELISA)

The levels of IL-1β, TNF-*α*, IL-6, GSH-Px, MDA, SOD, DA, dihydroxyphenylacetic acid (DOPAC) and homovanillic acid (HVA) levels in the cerebral cortex were determined, respectively, with ELISA kits according to the kit instructions (IL-1β, YJ712290, Shanghai Enzyme-linked Biotechnology Co., Ltd.; TNF-α and IL-6, RX302058R and RX302856R, QUANZHOU RUIXIN Biotechnology Co., Ltd., China; GSH-Px, 6141060130, Beijing Dongge Boye Biotechnology Co., LTD, China; MDA-S0131S and SOD-S0101S, Shanghai Biyuntian Bio-Technology Co., LTD, China; DA-m1002024, DOPAC-ml034074 and HVA-ml025114, Shanghai Enzyme linked Biotechnology Co., Ltd., China) on a microplate reader (American Hyperion MRIII type; Biotek Instruments Inc., Winooski, VT, USA). All samples were performed eight times in parallel. Detailed sample handling procedures refer to previous literature (Ning et al., 2022).

### 2.8 Hematoxylin–eosin (HE) and nissl staining

The hippocampus and striatum were prepared into continuous coronal sections, and dewaxed to water after conventional dewatering and embedding treatment. Dye with hematoxylin solution for 15 min, rinse with water, stain with alcohol eosin solution for 3 min, dehydrate and clear, seal with neutral gum, and observe under microscope. In addition, nissl staining is performed according to the Nissite kit (Methyl Violet method, 0409A14, Beijing Kangwei Century Biotechnology Co., LTD) instructions, observed under a microscope and photographed. All samples were performed six times in parallel.

### 2.9 Immunohistochemistry

The continuous coronal sections were dewaxed to water after tissue dehydration and embedding treatment. 3%H_2_O_2_, soaked for 10 min: pressure cooker repair (EDTA, pH 8.0); 5% BSA was blocked for 20 min, *α*-syn and TH (proteintech, 25859-1 and 10842-1, Wuhan Sanying Biotechnology Co., LTD, China) antibodies (1:50) were added and placed in a 37°C for 1 h, and secondary antibody (biotinized sheep anti-rabbit) was added to a 37°C for 20 min. Drip (SABC) at 37°C for 20 min; DAB was controlled under the microscope for 3 min; Anhydrous ethanol dehydrated transparent, neutral resin seal: microscope observation of positive expression of brown yellow, using the image analysis system (Beihang 4.0 version) for analysis. All samples were performed six times in parallel. PBS was used as a negative control instead of primary antibody.

### 2.10 Immunofluorescence

Dewaxing slices to water; 3% H_2_O_2_, soak for 10 min; Pressure cooker method for antigen repair (sodium citrate, pH 6.0): 5% BSA was blocked for 30 min and Beclin-1 (1:100, ab62557, Abcam, USA) and P62 (1:100, ab56416, Abcam, USA) antibodies (1:50) was added to 37°C for 1 h, and secondary antibody (biotinized sheep anti-rabbit) was added to 37°C for 20 min. Anti-extinguishing agent seal: the positive expression was red after microscope observation and photography, and the image analysis system (Zeiss, Oberkochen, Germany) was used for analysis. All samples were performed six times in parallel. PBS was used as a negative control.

### 2.11 Quantitative real-time polymerase chain reaction (qRT-PCR) analysis

The gene specific primers were demonstrated in Table 1. Total RNA was extracted from approximately 10 mg of mesocerebrum tissue by trizol. The RNA concentration was measured with spectrophotometry, and the RNA samples were stored at −80°C. Following the instructions in the Evo MMLV RT Mix kit with gDNA clean for qPCR Ver.2 (Cat: AG11728) manual, the RNA was reverse transcribed to cDNA. Next, RT-PCR was performed using the SYBR green premix pro taq HS qPCR kit (Cat:AG11701) to amplify and quantify IL-1β, TNF-*α*, IL-6, GSH-Px, ALB, SRC, ESR1, PTGS2, CDK1, CDK2, PARP1 mRNA levels and so on. The reaction conditions of qRT-PCR were as follows: pre-denaturation at 94°C for 2 min; denaturation at 94°C for 30 s; annealing at 58°C for 30 s; extension at 72°C for 1 min; 30 cycles; extension at 72°C for 8 min; and storage at −20°C. β-actin was used as internal reference control. Gene expression was calculated using the 2^-△△ct^ method (Xing et al., 2024; Yoav et al., 2024). All samples were performed six times in parallel.

**Table 1:** Primer sequences.

Gene	Primer sequences (5′-3′)		Gene	Primer sequences (5′-3′)	
ALB	F	GTGCTTGCAGAATTTCAGCCT	Cpa4	F	TCTGCGGCCGAGATAAATTCT
	R	TGTATCGAACCAGAATGGCGT		R	GAGAGGAAATGGTCTAGTCGGA
SRC	F	GCAGATTGTCAATAACACAGAGGG	Cdc6	F	TCCGTAAAGCGCTGGATGTT
	R	TGCCAAAGTACCACTCCTCAG		R	CGCTGGGTGATTTACATTCGG
PTGS2	F	CTGGGCCATGGAGTGGACTT	Lrr1	F	GGGAACCAGCTACAAGCTAAGA
	R	GAGGATACACCTCTCCACCG		R	CTCCTTTAGCCGGACAGTGG
CDK1	F	TTGTCACTCCCGACGAGTTC	Pbk	F	CCAGAGGGCTAAAGTACCTGC
	R	CAGCGTCACTACCTCGTGTG		R	TGGCAGAGAGACTCCTACATCA
EGFR	F	TCAACAACCAGAAGGGCCAA	Esco2	F	GGCGGTGTTCAGATGTACCA
	R	GCGGCGTAGTGTACGTTTTC		R	GCCAAACATGAAGCAATTCCTGA
CDK2	F	ATCCGGCTCGACACTGAGA	Tlr9	F	TGTGAGCTGAAGCCTCATGG
	R	GCAGCTTGACGATGTTAGGGT		R	GGTGGTGGATACGGTTGGA
PARP1	F	TCTGCACCAGCAGACAAACC	Spc24	F	ATGATCAAGGGCATCCACCAC
	R	ATGGCCTTTGCTTCGTCCTT		R	GTCACTGATGAACTTCGGCG
IL-1β	F	ATGCCACCTTTTGACAGTGATG	Nfkb1	F	AACAATGCCTTCCGGCTGA
	R	TGATGTGCTGCTGCGAGATT		R	GGCCTCCATCAGCTCTTTGAT
IL-6	F	CACTTCACAAGTCGGAGGCT	TNF	F	GATCGGTCCCCAAAGGGATG
	R	GAATTGCCATTGCACAACTCT		R	CCACTTGGTGGTTTGTGAGTG
TNF-α	F	CCCACGTCGTAGCAAACCAC	Gsta3	F	ACATGCCCCCTGAGGAGAAA
	R	TGAGATCCATGCCGTTGGC		R	TCCATGGCTCTTCAACACCTTTT
GSH-px	F	CATCCTGCCTTCTGTCCCTG	Pik3r1	F	GGCCTCCATCAGCTCTTTGAT
	R	CGCCATGGCAGTCTGTCTTA		R	TCAAACTCATGGAGACCTTTGCC
β-actin	F	TGGTGGGAATGGGTCAGAAG	Gprc5c	F	CAGAACAGAGCTACCAGGGG
	R	TGTAGAAGGTGTGGTGCCAG		R	CTGGTCTCTTTGCTGAGGCT

### 2.12 Western blot detection

The total protein was extracted from the nigral striatum of each group of mice by treating the brain tissue with protein lysate containing protease inhibitors and phosphoric acid inhibitors, and the protein content was determined by the BCA kit, and the protein was added into the sampling buffer and mixed, and the protein was denatured by heating at 100°C for 15 min. The protein samples were electrophoresed on 10% sodium dodecyl sulfate-polypropylene amine gel, transferred to PVDF membrane, and closed with 5% skimmed raw milk at room temperature for 1 h. α-syn and TH (1:1000, 25859-1 and 10842-1, proteintech, China), Beclin-1 (1:1000, ab62557, Abcam, USA), LC3B (1:1000, ab192890, Abcam, USA), P62 (1:1000, ab56416, Abcam, USA), P-PI3K (1:1000, 60225-1-Ig, proteintech, USA), PI3K (1:1000, ^#^13666 s, Cell Signaling Technology, USA), P-AKT (1:1000, 29163-1-AP, proteintech, China), AKT (1:1000, 10176-2-AP, proteintech, China) and GAPDH (1:1000, G9545, SIGMA, USA); P-CREB (1:1000, ^#^12133, SAB, USA), CREB (1:5000, 67927-1-Ig, proteintech, China), SRC (1:1000, 11097-1-AP, proteintech, China), BDNF (1:1000, ^#^32263, SAB, USA), PTGS2 (1:1000, 12375-1-AP, proteintech, China), mTOR (1:1000, 66888-1-Ig, proteintech, China) and P-mTOR (1:1000, 67778-1-Ig, proteintech, China) antibodies were added and incubated at 4°C overnight, then secondary antibodies were added and incubated at room temperature for 2 h. All samples were performed six times in parallel. The samples were detected by chemiluminescence reagent, developed and photographed by gel imager, and analyzed in grayscale by Image J software.

### 2.13 RNA sequencing and bioinformatics analysis in the mesencephalon tissue of PD model mice

OE Biotech Co., Ltd. (Shanghai, China) performed all RNA-sequencing and bioinformatics analyses. Differential analysis was performed according to Padj<0.05 and log2(fold change) < −1 or log2(fold change) >1 criteria to obtain differentially expressed genes, Using GraphPad Prism 8 software make volcanic diagram and through microscopic letter website (see Footnote 11) draw heat maps. All samples were performed three times in parallel.

### 2.14 Statistical analysis

Data were statistically analyzed using SPSS 17.0 software and expressed as mean ± SD. The data were analyzed using one-way analysis of variance (ANOVA) when the data were regularly distributed and the variance was elevated. ANOVA was used when the data were regularly distributed and the variance produced: nonparametric tests were used when the data were not regularly distributed. Statistical significance was accepted for *p* < 0.05.

## 3 Results

### 3.1 Study of the mechanism of Galangin against MPTP-induced PD mice by network analysis

**Figure 1:**
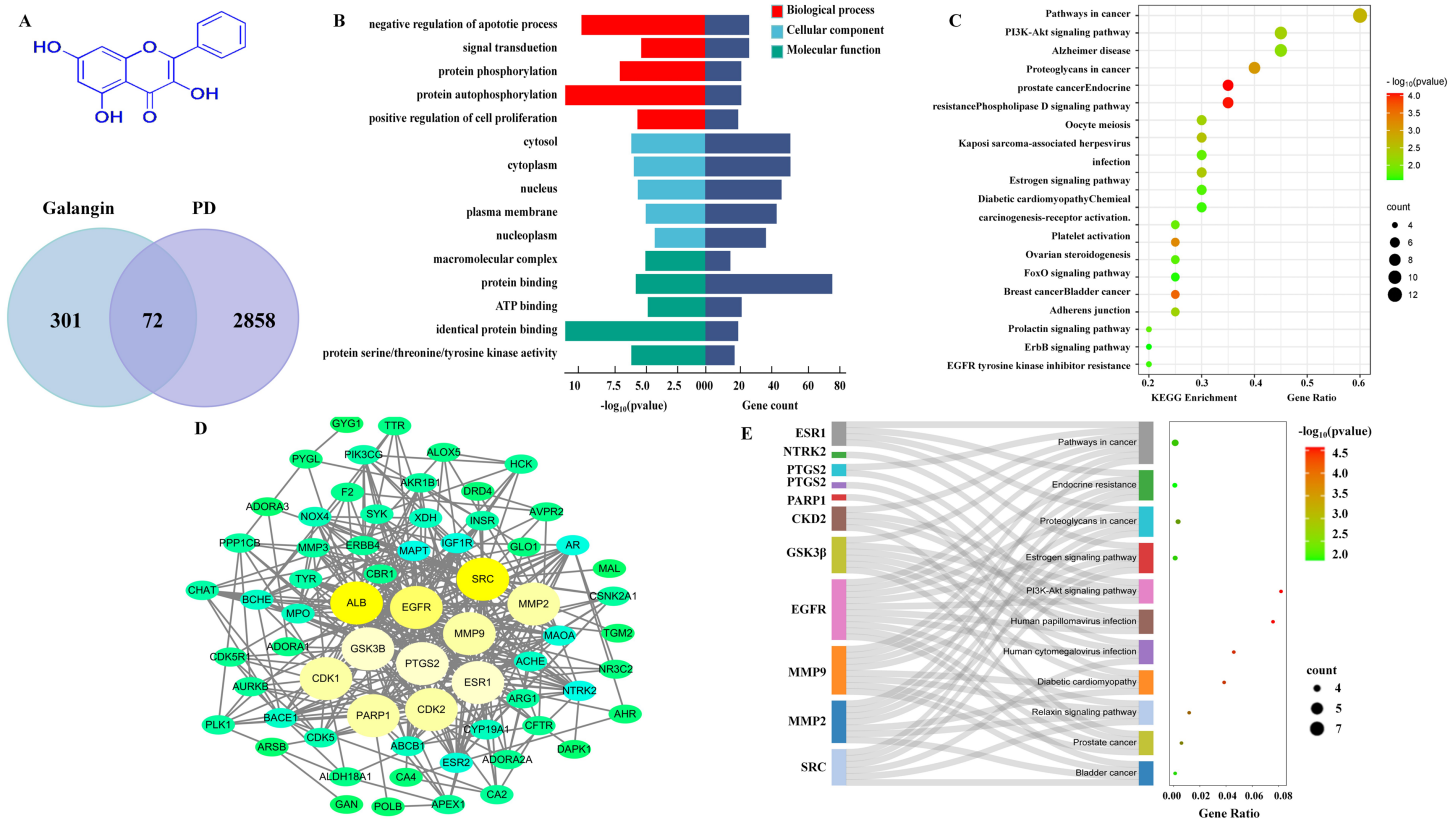
Network analysis predicts that there may be target-pathway interaction in Galangin anti-MPTP induced PD. **(A)** The structure of Galangin and Venn analysis on putative targets of Galangin and PD. **(B)** GO analysis of intersection targets. **(C,E)** The pathway enrichment analysis was performed related to intersection targets. **(D)** PPI network showed the correlation between PD-related gene targets, and the larger the circle, the greater the degree value. The top 10 core targets were ALB, SRC, PTGS2, EGFR, GSK3β, ESR1, MMP9, PARP1, CDK1, and CDK2. MPTP, N-methyl-4-phenyl-1,2,3,6-tetrahydropyridine; PD, Parkinson’s disease; PPI, protein–protein interaction; ALB, albumin; SRC, Sample Rate Convertor; PTGS2, Prostaglandin Endoperoxide Synthase 2; EGFR, Epidermal growth factor receptor; GSK3β, Glycogen synthase kinase 3β; ESR1, Estrogen Receptor 1; MMP9, matrix metalloprotein; PARP1, Poly ADP-ribose polymerase 1; CDK1, cyclin dependent kinase 1; CDK2, cyclin dependent kinase 2.

Network analysis was employed to predict the potential target-pathway interactions associated with the protective effect of Galangin against PD. Firstly, through an extensive search of the TCMSP, Swiss Target Prediction and PharmMapper databases, 373 targets of Galangin were identified. Meanwhile, 2,858 genes related to PD were identified from the DisGeNET and Genecards databases, and 72 shared targets between Galangin and PD targets. The structure of Galangin and the Venn diagram of intersecting targets are shown in Figure 1A. Table 2 shows the top 10 targets, ALB, SRC, ESR1, EGFR, GSK3β, PTGS2, MMP9, PARP1, CDK2 and CDK1, which were referred to as core proteins. The study confirmed that they can interact with different proteins and occupy a crucial position in the network, which can provide a basis for additional research. In addition, a total of 194 biological processes, 57 cellular components, and 69 molecular functions were acquired. The pathways with the top 10 enriched genes were selected for display, as depicted in Figure 1B. The targets acted on the negative regulation of apoptotic process and protein phosphorylation, and protein autophosphorylation, response to xenobiotic stimulus, cellular response, and peptidyl-serine phosphorylation played a role. Meanwhile, these target functions were associated with protein binding, ATP binding, identical protein binding, and enzyme binding. As shown in Figure 1C, pathways in cancer, PI3K-AKT signaling pathways, and chemokine signaling pathways were mainly related to the effects of Galangin against PD. The noninteracting targets were eliminated, and the interactions between the remaining targets are shown in Figure 1D by a PPI network. Finally, we mapped 10 predicted targets onto 10 corresponding pathways, as shown in Figure 1E.

**Table 2:** Top 10 potential targets of Galangin for PD treatment.

Target	Degree	Betweenness	Closeness
ALB	39	0.152425238	0.698924731
SRC	33	0.155089697	0.650000000
ESR1	32	0.104395481	0.650000000
EGFR	31	0.067632251	0.643564356
GSK3β	30	0.104534353	0.643564356
PTGS2	30	0.056273119	0.625000000
MMP9	23	0.023426488	0.580357143
PARP1	20	0.037831610	0.565217391
CDK2	20	0.026283225	0.550847458
CDK1	20	0.036521346	0.570175439

### 3.2 Galangin treatment improved motor coordination of PD mice

**Figure 2:**
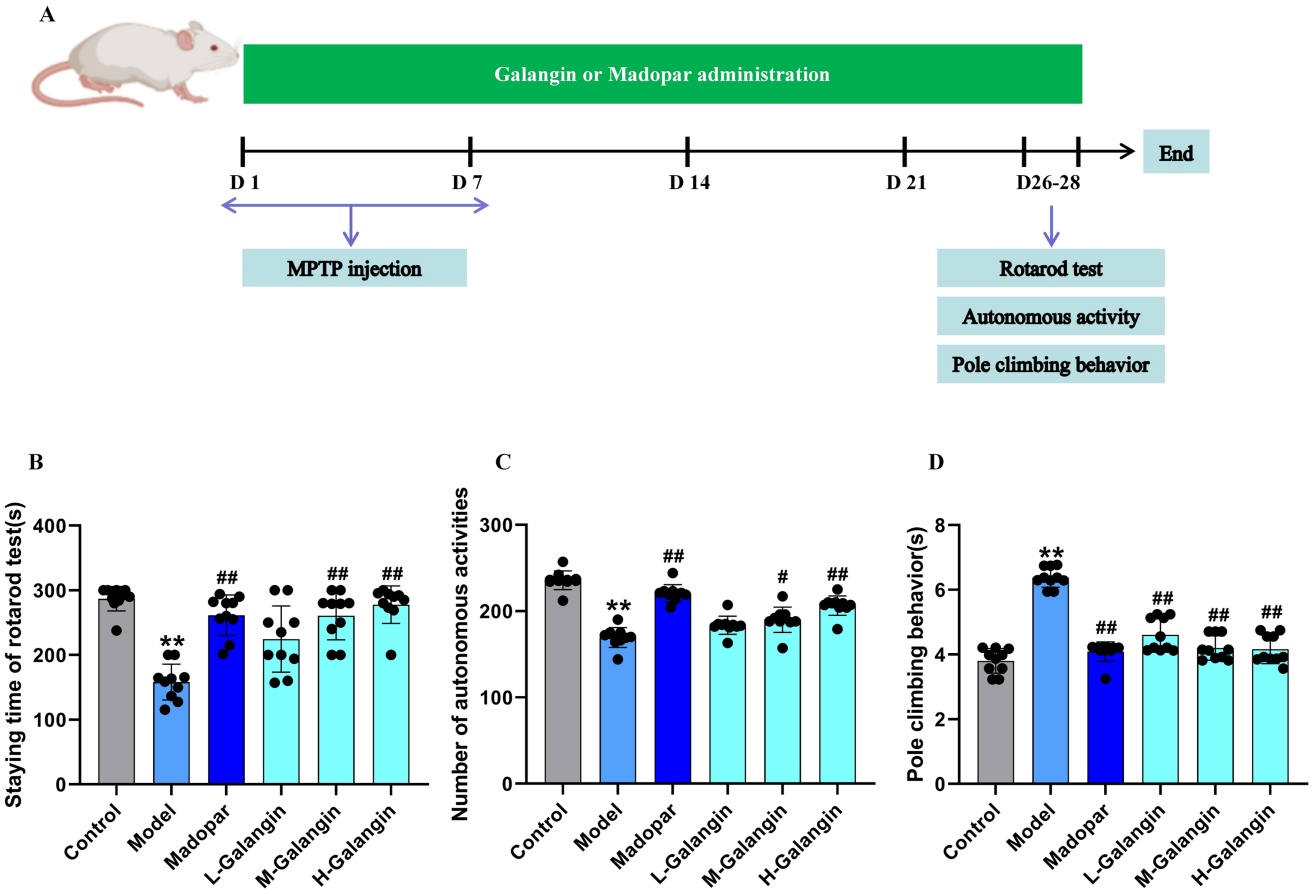
Galangin treatment improves motor coordination in PD mice. **(A)** Galangin administration design and behavioral testing schedule in mice. **(B)** Staying time of rotarod test in PD mice. **(C)** The autonomous activity in PD mice. **(D)** The pole climbing behavior test in PD mice. *n* = 10 in each group; ^**^p < 0.01 vs. control group; ^#^*p* < 0.05, ^##^*p* < 0.01 vs. model group; The *F*-values for **(B–D)** are 6.852, 262.950 and 34.456, respectively. PD, Parkinson’s disease; L-Galangin, low dose of Galangin; M-Galangin, medium dose of Galangin; H-Galangin, high dose of Galangin.

To evaluate the effect of Galangin on motor coordination in PD mice, the rotarod, autonomous activity and pole climbing tests were measured. The process and results of the behavior experiment are shown in Figures 2A–D. The motor coordination ability of mice in the MPTP model group significantly decreased compared to that in the control group (*p* < 0.01). The specific manifestations are as follows: the staying time in the rotarod test (Figure 2B) and the count of autonomous activities (Figure 2C) were increased in the madopar-, M-Galangin-, and H-Galangin-treated mice compared to those in the PD model group (*p* < 0.05 or *p* < 0.01). Additionally, the pole climbing time was reduced in the madopar- and all doses of Galangin-treated mice compared to the PD model mice (*p* < 0.05 or *p* < 0.01, Figure 2D). In conclusion, these results demonstrated that Galangin enhanced the motor coordination and endurance of PD model mice.

### 3.3 Galangin ameliorates MPTP-induced brain inflammation and oxidative stress damage

**Figure 3:**
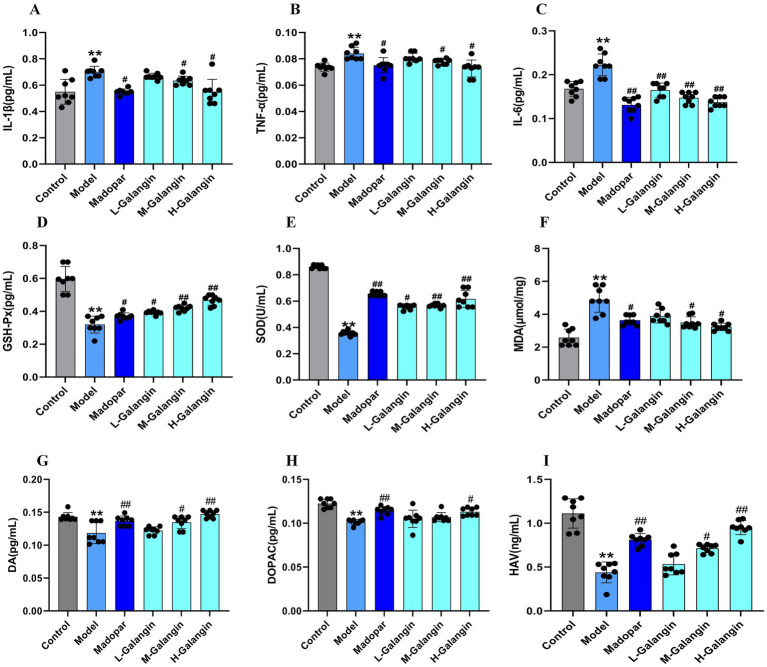
Galangin reduces brain inflammatory injury and decreases oxidative stress. Expressed protein levels of **(A)** IL-1β, **(B)** TNF-*α*, **(C)** IL-6, **(D)** GSH-Px, **(E)** SOD, **(F)** MDA, **(G)** DA, **(H)** DOPAC, and **(I)** HVA were determined by ELISA (*n* = 8). ^*^p < 0.05, ^**^p < 0.01 vs. control group; ^#^*p* < 0.05, ^##^*p* < 0.01 vs. model group. The *F*-values for A to I are 4.497, 4.861, 4.765, 5.132, 5.034, 6.261, 12.364, 393.057, and 179.145, respectively. IL-1β, interleukin-1β; TNF-α, tumor necrosis factor-α; IL-6, Interleukin-6; GSH-Px, glutathione peroxidase; SOD, Superoxide dismutase; MDA, Malondialdehyde; H-Galangin, high dose of Galangin; DA, Dopamine; DOPAC, dihydroxyphenylacetic acid and HVA, homovanillic acid.

Previous studies have shown that brain neuroinflammation is one of the crucial pathogeneses of PD (Chrysoula et al., 2020). The resistance to brain injury induced by Galangin was assessed by measuring the levels of IL-1β, TNF-*α*, IL-6 and GSH-Px, SOD, MDA using ELISA (Figures 3A–F). The present data indicated that compared with the control mice, the levels of IL-1β, TNF-α, IL-6 in the brain tissue of PD model mice increased significantly, however, the situation for GSH-Px was the opposite (*p* < 0.01). Moreover, the levels of IL-1β, TNF-α, IL-6 and MDA in the Galangin groups were significantly decreased compared with those in the PD model group, while the levels of GSH-Px and SOD were significantly elevated (*p* < 0.05 or *p* < 0.01). Additionally, MPTP, after crossing the blood–brain barrier, damages the dopaminergic neurons in the substantia nigra pars compacta (SNc) and the striatum, leading to a decrease in DA levels, which in turn causes PD-like behavioral abnormalities and neuropathological changes (Episcopo et al., 2013). Our study found that compared to the control group, the levels of DA and its metabolites DOPAC and HVA in the striatum of the PD model group mice were significantly decreased. Compared to the PD model group, the levels of DA and its metabolites DOPAC and HVA in the Madopar group and the high-dose Galangin group were significantly increased (*p* < 0.05 or *p* < 0.01, Figures 3G–I). Collectively, these results indicate that Galangin increases DA level and decreases oxidative damage in the striatum of mice induced by MPTP. Thus, Galangin could be an effective agent against oxidative injury for the treatment of PD.

### 3.4 Effect of Galangin on histopathological changes in the brain of mice with PD

**Figure 4:**
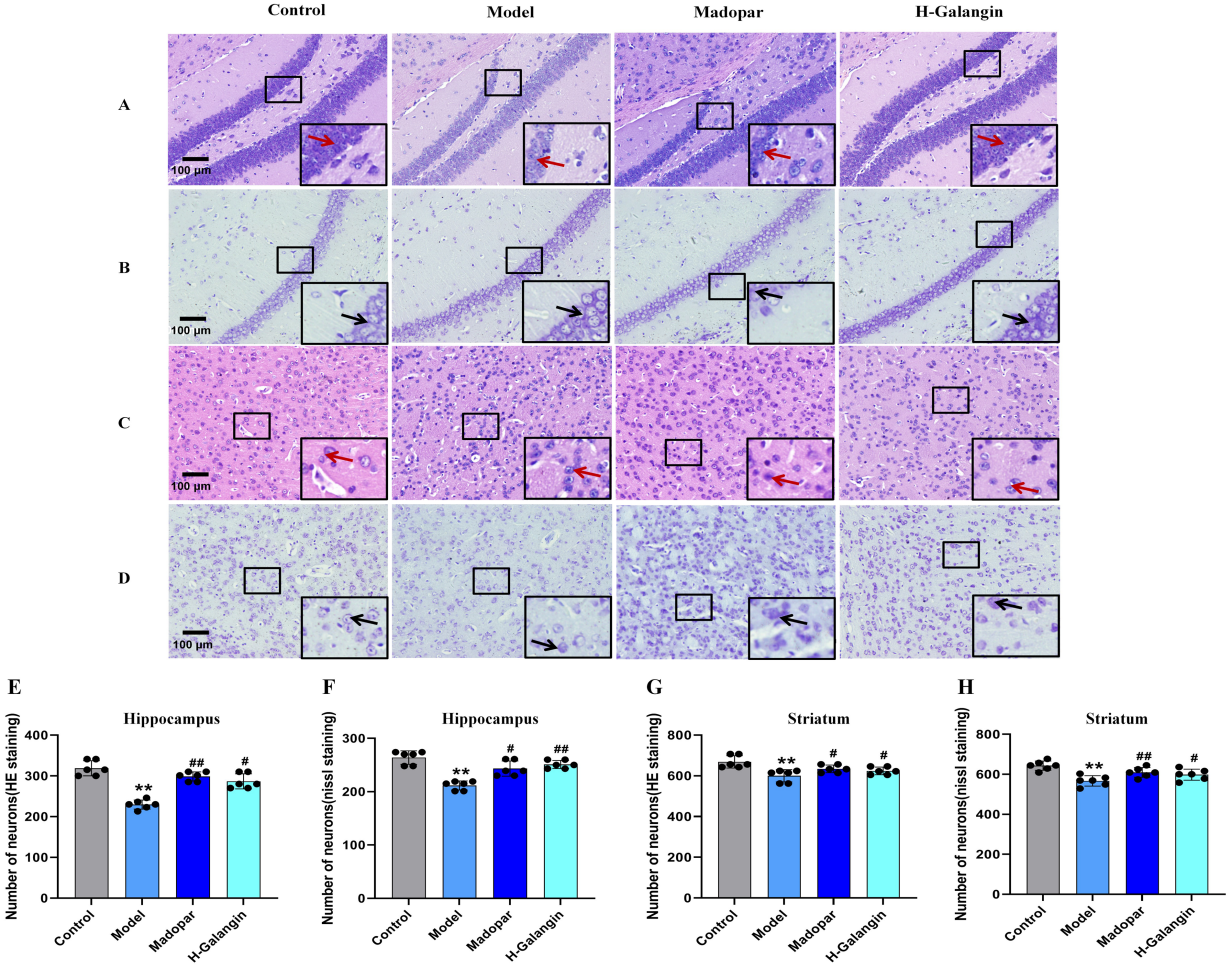
Histopathology of hippocampus and striatum of mice in each group (*n* = 6, ×200). **(A)** Represents the HE staining of the hippocampus; **(B)** represents the nissl staining of hippocampus; **(C)** represents HE staining of the striatum; **(D)** represents the nissl staining of the striatum. **(E)** represents the number of neurons in hippocampus by HE staining; **(F)** represents the number of neurons in hippocampus by nissl staining; **(G)** represents the number of neurons in striatum by HE staining; **(H)** represents the number of neurons in striatum by nissl staining. ^**^p < 0.01 vs. control group; ^#^*p* < 0.05, ^##^*p* < 0.01 vs. model group. The *F*-values for E, F, G and H are 16.410, 9.701, 3.078, and 10.909, respectively. H-Galangin, high dose of Galangin. Black arrows indicate the nissl bodies and red arrows indicate neurons.

The results of HE staining (Figures 4A,C,E,G) and nipponite staining (Figures 4B,D,F,H) showed that the number of neurons in the CA1 area of the hippocampus and striatum in the control group was increased, with a graphic or oval morphology, neat arrangement, clear hierarchy, and abundant nidus; compared with the control group, the number of neurons in the CA1 area of the hippocampus and striatum in the model group was significantly reduced (*p* < 0.01), the morphology was triangular or irregular polygonal, the number of free cells were increased and arranged in a disordered manner, the hierarchy was unclear, the cytosol was solidified and profoundly stained, and the number of nipponite bodies was less (*p* < 0.01); compared with the model group, the neuronal pathological changes in the hippocampal CA1 area and striatal of the madopar and H-Galangin groups showed different degrees of attenuation, with tiny cell gaps, higher number of cells, orderly arrangement, and a significant increase in the number of nipponite bodies (*p* < 0.05 or *p* < 0.01). The above results suggest that Galangin could reduce the degree of neuronal pathology caused by MPTP.

### 3.5 Galangin reduces *α*-syn deposition and promotes TH expression

**Figure 5:**
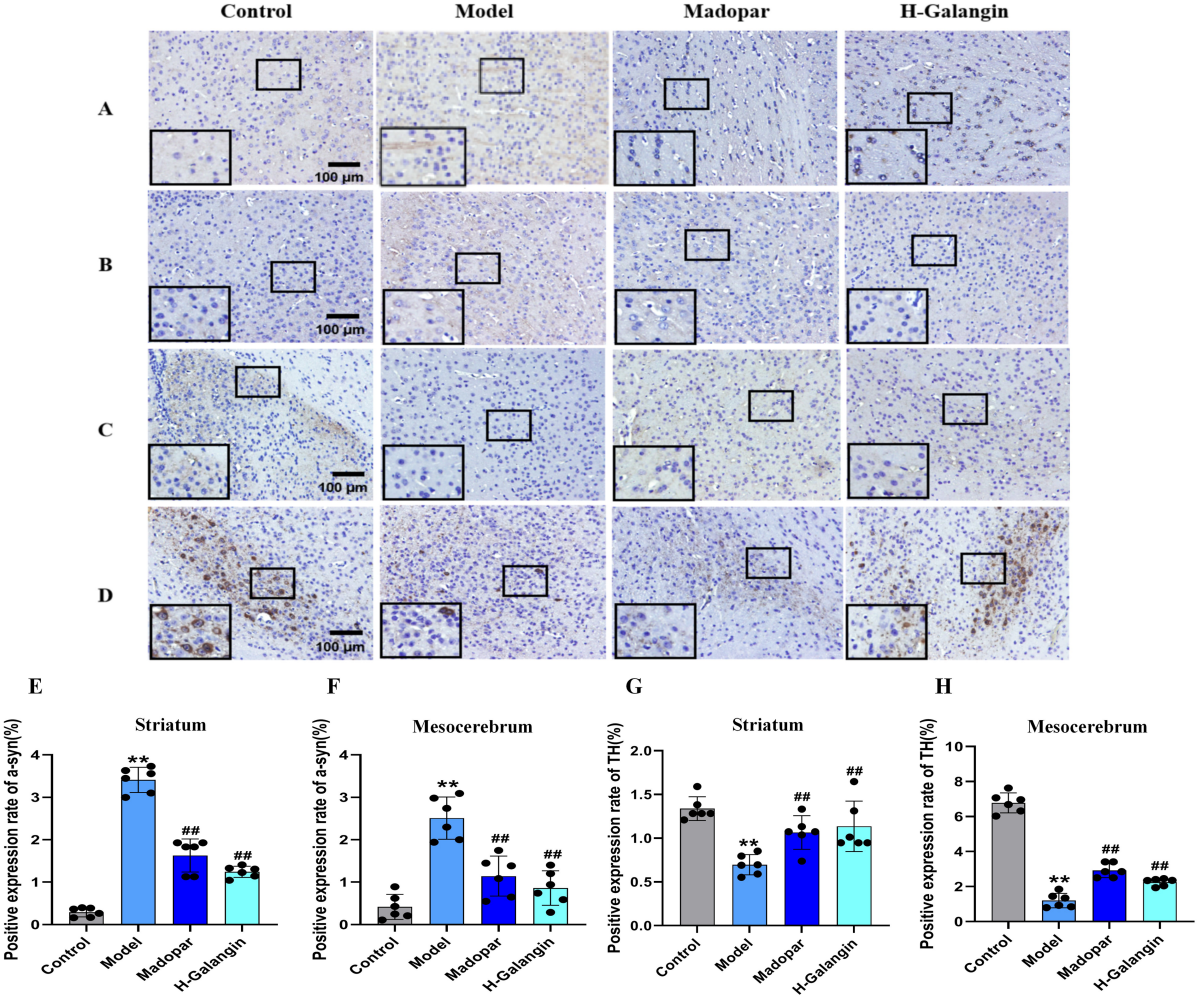
The expression of α-syn and TH in striatum and mesocerebrum of each group was detected by immunohistochemistry (*n* = 6, ×200). **(A)** Represents the α-syn expression of the striatum; **(B)** represents the α-syn expression of the mesocerebrum; **(C)** represents the TH expression of the striatum; **(D)** represents the TH expression of the mesocerebrum; **(E)** represents the changes in α-syn expression of striatum in each group; **(F)** represents the changes in α-syn of mesocerebrum in each group; **(G)** represents the changes in TH expression of striatum in each group; **(H)** represents the changes in TH expression of mesocerebrum in each group. ^**^p < 0.01 vs. control group; ^#^*p* < 0.05, ^##^*p* < 0.01 vs. model group. The *F*-values for **(E–H)** are 58.562, 8.582, 16.092 and 131.747, respectively. α-syn, α-synuclein; TH, Tyrosine hydroxilase; H-Galangin, high dose of Galangin.

As shown in Figure 5, compared with the control group, the expression of α-syn in the striatum (Figures 5A,E) and mesocerebrum (Figures 5B,F) of mice in the model group was significantly increased, while the expression of TH was significantly attenuated (*p* < 0.01); compared with the model group, the expression of α-syn in the striatum and mesocerebrum of mice in the madopar- and Galangin-treated groups was significantly down-regulated, while the expression of TH in the striatum (Figures 5C,G) and mesocerebrum (Figures 5D,H) was significantly enhanced (*p* < 0.05 or *p* < 0.01).

### 3.6 Galangin alleviates autophagy and activates PI3K/AKT pathway

**Figure 6:**
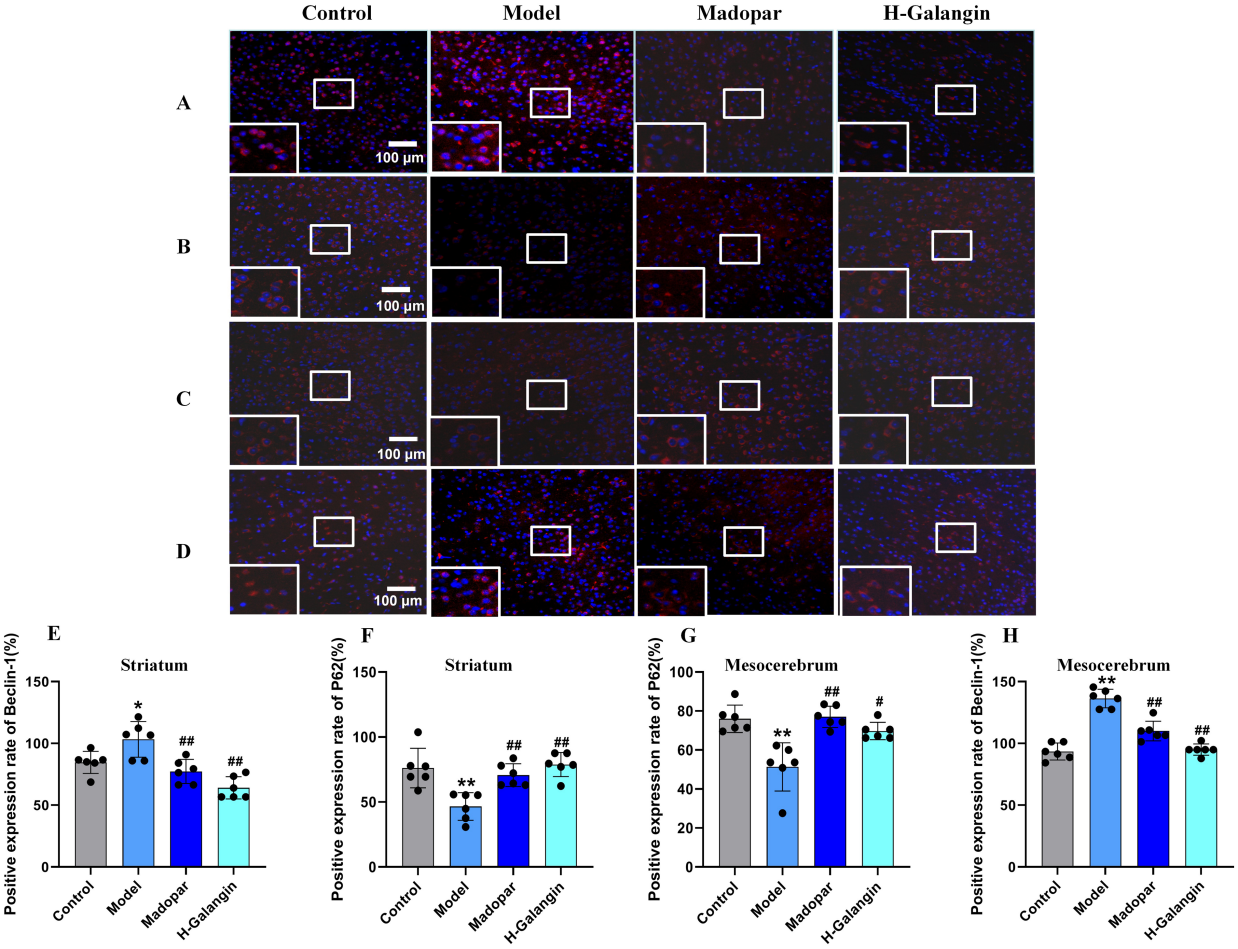
The expression of Beclin-1 and P62 in striatum and mesocerebrum of each group was detected by immunofluorescence (*n* = 6, ×200). **(A)** Represents the Beclin-1 expression of the striatum; **(B)** represents the P62 expression of the striatum; **(C)** represents the P62 expression of the mesocerebrum; **(D)** represents the Beclin-1 expression of the mesocerebrum; **(E)** represents the changes in Beclin-1 expression of striatum in each group; **(F)** represents the changes in P62 expression of striatum in each group; **(G)** represents the changes in P62 expression of mesocerebrum in each group; **(H)** represents the changes in Beclin-1 expression of mesocerebrum in each group; ^*^p < 0.05, ^**^p < 0.01 vs. control group; ^#^*p* < 0.05, ^##^*p* < 0.01 vs. model group. The *F*-values for **(E–H)** are 4.283, 4.283, 4.280, and 11.320, respectively. H-Galangin, high dose of Galangin.

**Figure 7:**
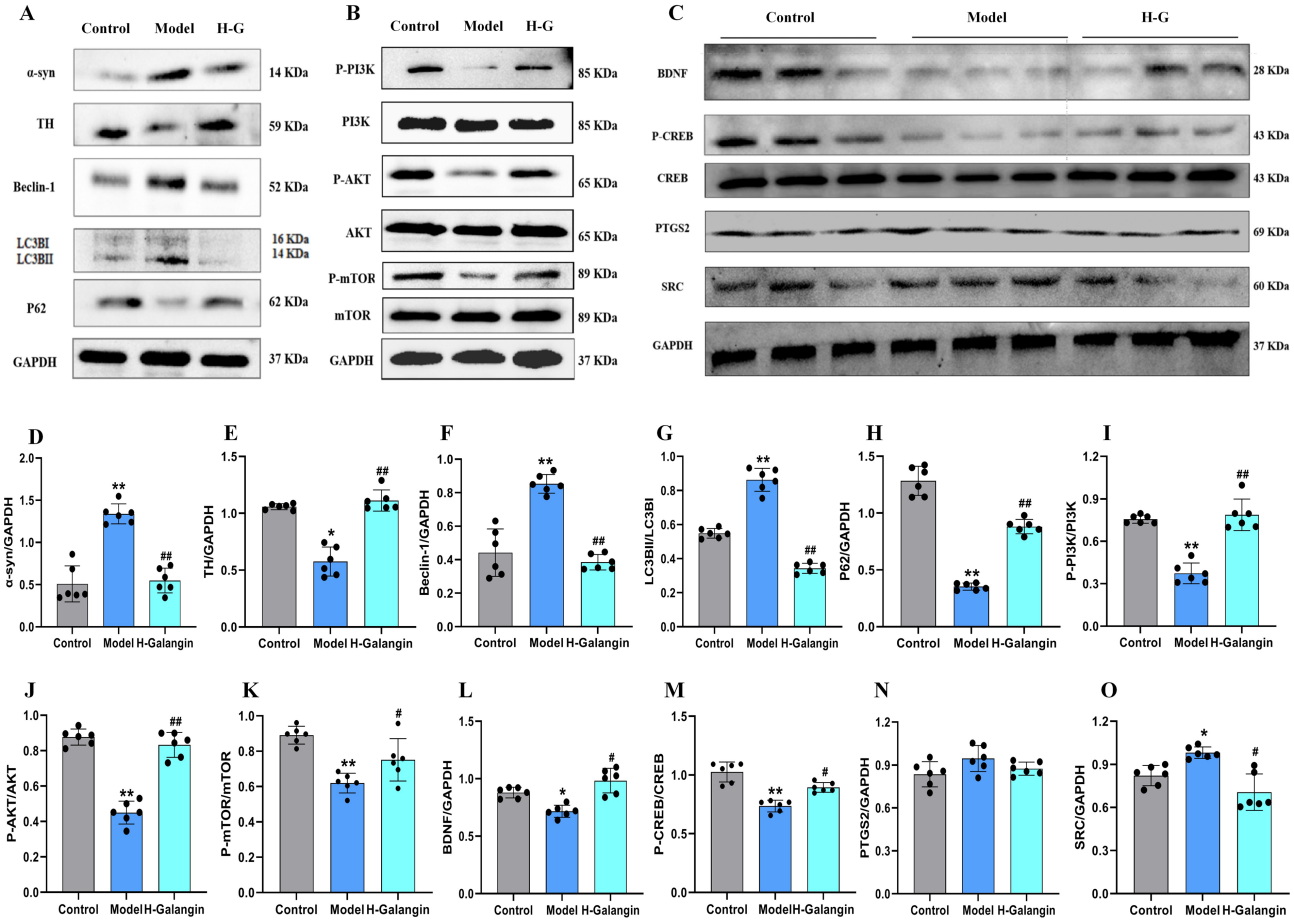
Galangin alleviated autophagy and activated PI3K/AKT signaling pathway in PD mice. **(A)** Protein levels of α-syn, TH, Beclin-1, LC3BII, P62, **(B)** protein levels of P-PI3K, P-AKT, P-mTOR, and **(C)** protein levels of BDNF, P-CREB, PTGS2 and SRC in mesocerebrum were determined by western blotting. Quantitative analyses of α-syn **(D)**, TH **(E)**, Beclin-1 **(F)**, LC3BII **(G)**, P62 **(H)**, P-PI3K **(I)**, P-AKT **(J)**, P-mTOR **(K)**, BDNF **(L)**, P-CREB **(M)**, PTGS2 **(N)** and SRC **(O)** by e-Blot software. *n* = 6, ^*^p < 0.05, ^**^p < 0.01 vs. control group; ^#^*p* < 0.05, ^##^*p* < 0.01 vs. model group. The *F*-values for **(A–C)** are α-syn (48.623), TH (32.490), Beclin-1 (15.752), LC3BII (133.073), P62 (355.598), P-PI3K (184.939), P-AKT (41.508), P-mTOR (20.195), BDNF (11.111), P-CREB (14.550) and PTGS2 (2.400) and SRC (7.697), respectively. PD, Parkinson’s disease; α-syn, α-synuclein; TH, tyrosine hydroxilase; H-Galangin, high dose of Galangin; PI3K, Phosphatidylinositol 3-Kinase; AKT, Protein Kinase B; PTGS2, prostaglandin-endoperoxide synthase 2 Gene; BDNF, brain-derived neurotrophic factor; CREB, cAMP-response element binding protein; SRC, Sample Rate Convertor.

The PI3K/AKT signaling pathway can be activated by various types of cell stimulation or toxic injury, and has basic cellular functions such as regulating cell proliferation, apoptosis, and differentiation. Studies have shown that activation of PI3K/AKT signaling pathway inhibits autophagy, which can reduce nerve function injury and play a neuroprotective role (Guo et al., 2015). To verify the effect of Galangin on the PI3K/AKT signaling pathway and autophagy after MPTP-induced neuron injury, IF (Figures 6A–H) and WB (Figures 7A–O) analyses were used to evaluate the protein expression levels of *α*-syn, TH, P-PI3K, P-AKT, P-mTOR, Beclin-1, LC3BII/LCBI, P62 in the mesocerebrum of the brain at 28 days after treating. The results suggested that the protein levels of TH, P-PI3K, P-AKT and P62 was significantly downregulated in the PD model group compared to that in the control group (*p* < 0.05 or *p* < 0.01), and *α*-syn, Beclin-1 and LC3BII/LCBI were significantly upregulated (*p* < 0.05 or *p* < 0.01). Moreover, the protein levels of α-syn, SRC, Beclin-1 and LC3BII/LCBI were decreased, and the protein levels of TH, BDNF, P-CREB, P-PI3K, P-AKT, P-mTOR and P62 protein were increased by Galangin treatment (*p* < 0.05 or *p* < 0.01). These results indicated that Galangin alleviates MPTP-induced autophagy and activates the protein levels of P-PI3K/PI3K and P-AKT/AKT to protecting neurons in PD mice.

### 3.7 Mesocerebrum transcriptome analysis

**Figure 8:**
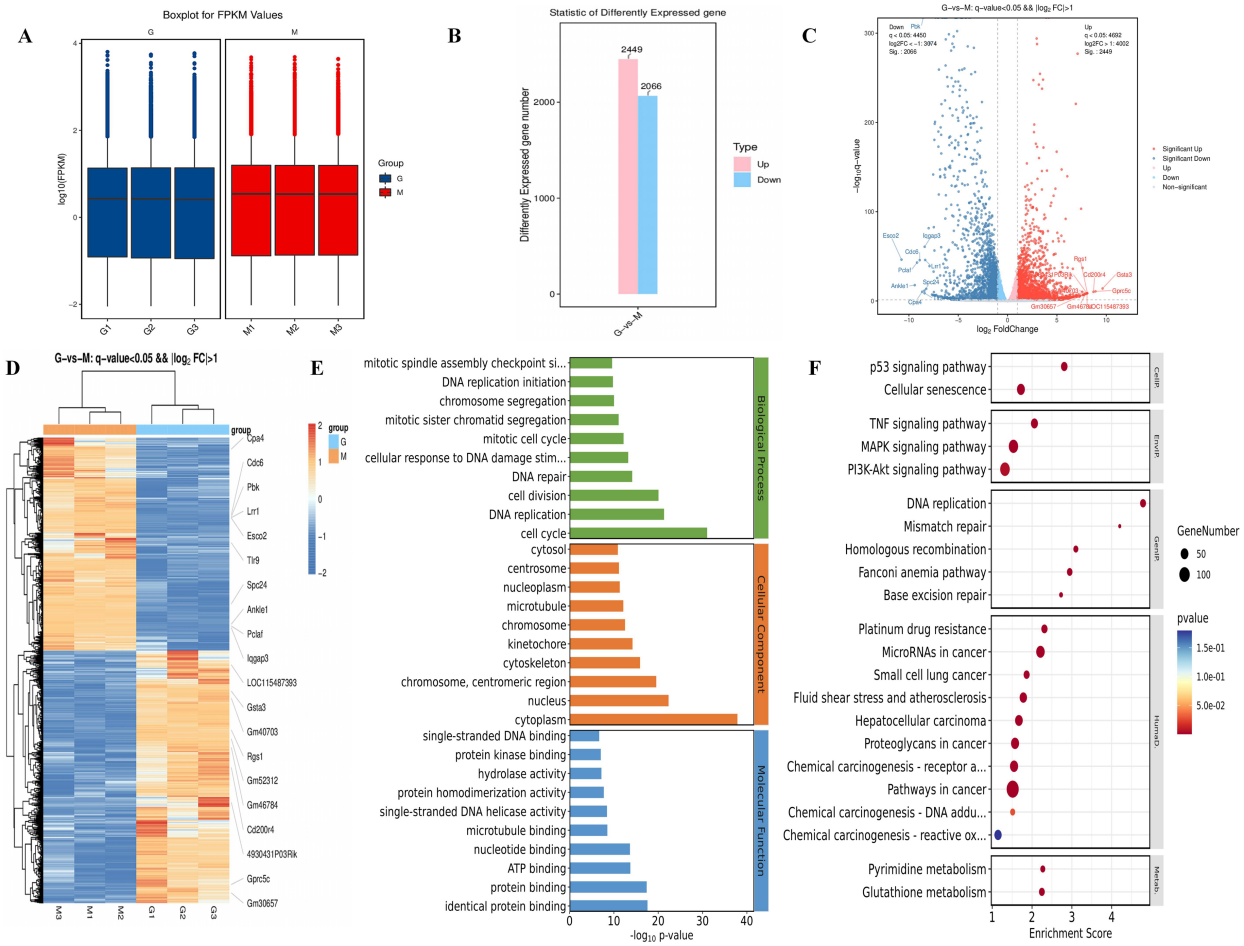
Transcriptomic analysis and differential gene validation results in the mesocerebrum of mice. **(A)** The FPKM boxplot group G and Group M. **(B)** Statistic of differently expressed gene in group G and Group M. **(C)** Differential gene volcano map of two groups of group G and group M. **(D,E)** show the KEGG and GO analysis result diagrams for the group G and group M, respectively. **(F)** Differential gene heat maps of group G and group M. Red, up-regulated gene; Blue, down-regulated gene; group G, H-Galangin group; Group M, model group.

The heat map and volcano map of differentially expressed genes showed the distribution of differentially expressed genes in the mesocerebrum tissue of the two groups of mice. There were 4,515 differentially expressed genes in H-Galangin and model mice, including 2,449 up-regulated genes and 2066 down-regulated genes (*p* < 0.05, Figures 8A–C). Compared with the model group, the expression of Cpa4, Cdc6, pbk, Lrr1, Esco2, Tlr9, spc24, Ankle1, Pclaf, lqgap3 and other genes in the mesocerebrum of H-Galangin group was significantly down-regulated. Expressions of LOC115487393, Gsta3, Gm40703, Rgs1, Cd200r4 and other genes were significantly up-regulated (Figures 8D–F).

### 3.8 Galangin changed the transcriptional levels of the candidate genes

**Figure 9:**
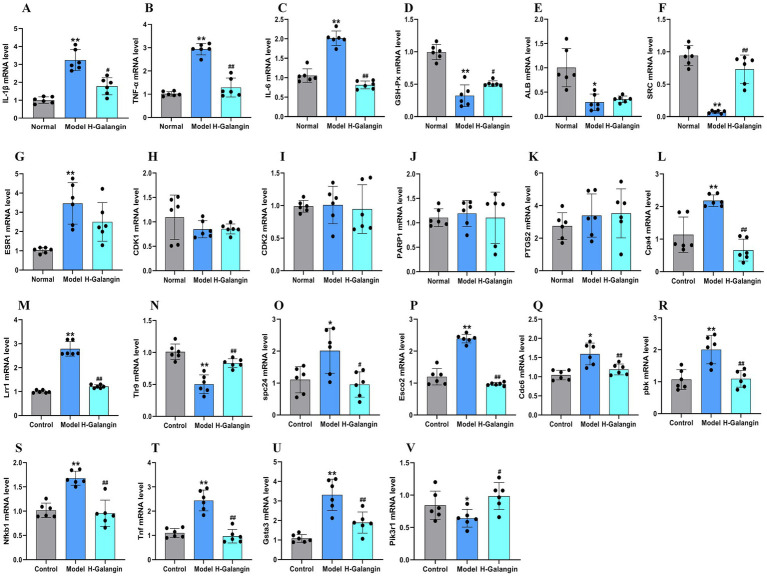
RT-qPCR analysis of differentially expressed genes in the mesocerebrum of each group mice. *n* = 6, ^*^p < 0.05, ^**^p < 0.01 vs. control group; ^#^*p* < 0.05, ^##^*p* < 0.01 vs. model group. The *F*-values for **(A–V)** are **(A)** (IL-1β, 244.893), **(B)** (TNF-α, 6.800), **(C)** (IL-6, 13.980), **(D)** (GSH-Px, 29.913), **(E)** (ALB, 12.895), **(F)** (SRC, 38.801), **(G)** (ESR1, 3.219), **(H)** (CDK1, 0.776), **(I)** (CDK2, 0.094), **(J)** (PARP1, 0.128), **(K)** (PTGS2, 26.508), **(L)** (Cpa4, 9.184), **(M)** (Lrr1, 67.731), **(N)** (Tlr9, 9.630), **(O)** (spc24, 2.557), **(P)** (Esco2, 1007.165), **(Q)** (Cdc6, 9.712), **(R)** (pbk, 9.482), **(S)** (Nfkb1, 18.542), **(T)** (Tnf, 13.589), **(U)** (Gsta3, 20.973), and **(V)** (Pik3r1, 9.482), respectively. IL-1β, interleukin-1β; TNF-α, tumor necrosis factor-α; IL-6, Interleukin-6; GSH-Px, glutathione peroxidase; ALB, albumin; SRC, Sample Rate Convertor; ESR1, Estrogen Receptor 1; PTGS2, Prostaglandin Endoperoxide Synthase 2; CDK1, cyclin dependent kinase 1; CDK2, cyclin dependent kinase 2; PARP1, Poly ADP-ribose polymerase 1; Cpa4, carboxypeptidase a4; Lrr1, leucine rich repeat protein 1; Tlr9, toll-like receptor 9; spc24, spindle pole component 24; Nfkb1, nuclear factor kappa B subunit 1; Tnf, Tumor necrosis factor; Gsta3, glutathione S-transferase alpha 3; Pik3r1, phosphoinositide-3-kinase regulatory subunit 1; Esco2, establishment of sister chromatid cohesion N-acetyltransferase 2; Cdc6, cell division cycle 6; pbk, PDZ binding kinase; H-Galangin, high dose of Galangin.

Galangin can alter the expression of predicted core proteins. Nevertheless, how it affects these candidate proteins remains unclear. To further clarify the molecular mechanism by which Galangin regulated protein expression, we examined the mRNA expression of IL-1β, TNF-*α*, IL-6, GSH-Px, ALB, SRC, ESR1, PTGS2, CDK1, CDK2 and PARP1 in mesocerebrum according to the results of network analysis. The qRT-PCR results showed that after treating PD model mice with H-Galangin (100 mg/kg) for 28 days, the mRNA levels of IL-1β, TNF-α, IL-6, SRC, PTGS2 were upregulated except ESR1, GSH-Px and ALB in the mesocerebrum of the PD model mice compared with the control mice (*p* < 0.05). There was no significant difference in CDK1, CDK2, PARP1 and ESR1 expression in PD model mice compared to control mice after treating Galangin. Moreover, these results indicated that Galangin downregulated the levels of IL-1β, TNF-α, IL-6, SRC, and PTGS2 mRNA compared with those of PD model mice (*p* < 0.05 or *p* < 0.01), However, it did not affect ALB, CDK1, CDK2, ESR1, PARP1 (Figures 9A–K). Consequently, these data suggested that Galangin decreased the transcript levels of IL-1β, TNF-α, IL-6, SRC and PTGS2 in PD model mice.

In order to further verify the expression level of differential genes by transcriptome sequencing, the key genes of differentially expressed genes in the midbrain of two groups of mice were verified by RT-qPCR. Compared with the model group, mRNA expression of Gpc5c genes in mesocerebrum of the H-Galangin treated group was significantly up-regulated (*p* < 0.05 or *p* < 0.01). In addition, the most significantly down-regulated gene mRNA was Cpa4, Lrr1, Tlr9, spc24, nfkb1, Tnf, Gsta3, Pik3r1, Esco2, Cdc6 and pbk (*p* < 0.05 or *p* < 0.01), which was consistent with the trend of transcriptome sequencing (Figures 9L–V).

## 4 Discussion

Currently, research on the use of Galangin in treating PD is limited, and our study remains an early exploratory investigation. Following the 4R principles, we adopted a dual-dose (high and low) administration approach. Our experimental results showed that Galangin improved motor coordination abilities in mice with PD during the behavioral tests. These results can be a supplement for the experimental study of Galangin anti-PD (Chen et al., 2017; Chen et al., 2022), and support the results of our network analysis and molecular docking that Galangin may be a candidate for the PD agent. MPTP-induced experimental model of PD is a representative research model, the mechanism of which is the entry of MPTP into the body. It is converted to MPP^+^ under the action of monoamine oxidase to produce symptoms similar to PD (Jackson-Lewis and Przedborski, 2007). MPP^+^ production will lead to ATP production disorder, intracellular Ca^2+^ level increase and reactive oxygen species production, causing dopaminergic neuron death (Episcopo et al., 2013). Studies have shown that Galangin is useful for treating PD (Kilic et al., 2019; Zeng et al., 2015; Huang et al., 2016). It is difficult to accurate insight into the potential pharmacological mechanisms by which Galangin improves motor coordination using traditional methods. Consequently, we used network analysis and molecular docking to predict and validate the relevant targets and possible mechanisms. Additionally, we employed transcriptome sequencing to construct and analyze a target network, identifying multiple drug targets. Enrichment and functional analyses were conducted to further elucidate Galangin’s mechanisms of action.

Research has confirmed that the key targets of Galangin against liver cancer were SRC, ESR1, MMP9, CDK4, CCNB1, MMP2, CDK2, CDK1, CHEK1 and PLK1 (Li et al., 2024). And the other research found that Galangin administration significantly suppressed the prominent enhancement of PTGS2 induced by RSL3 in HT1080 cells (Chen et al., 2022). Based on the analysis of our network analysis in Galangin with PD, we successfully found that SRC, ESR1 and PTGS2 are important regulators of Galangin in PD. The lignans of S. chinoea can down-regulate the expression of SRC protein in SW1353 cells induced by IL-1β, and then regulate the inflammatory response in the body (Min et al., 2021; Li et al., 2021). PTGS2 is an up-regulated gene, and the expressions of PTGS2, nf-κb increased in MCAO rats, and the specific expression of PTGS2 is decreased, which can inhibit the nf-κb signaling pathway, inhibit cell apoptosis, and promote the proliferation, migration and angiogenesis of endothelial progenitor cells (Zhou et al., 2019). ESR1 is mainly expressed in endothelial cells, vascular smooth muscle cells and macrophages, and plays an influential role in the physiology and function of blood vessel walls. Mutations in the ESR1 gene may lead to cerebral infarction (Gao et al., 2014). We hypothesize that Galangin modulates the mRNA levels of SRC, ESR1 and PTGS2, thereby improving motor coordination abilities. However, we expect that multiple mechanisms contribute to this function. KEGG pathway enrichment analysis predicted that Galangin ameliorates neuroinflammation by regulating multiple pathways of which regulation of the PI3K/AKT signaling pathway is critical. The PI3K/AKT signaling pathway has multiple roles, closely related to cell proliferation, apoptosis, oxidative stress, and inflammatory reaction (Ji and Wang, 2019; Manning and Toker, 2017). However, it is not clear whether Galangin is interlinked with this signaling pathway in motor coordination after MPTP successfully constructed PD model. After experimental verification, it was found that PI3K/AKT signaling pathway is disrupted in mice with MPTP injury. In contrast, Galangin dose-dependently restored PI3K/AKT signaling pathway activation, which is critical for normal brain functioning.

**Figure 10:**
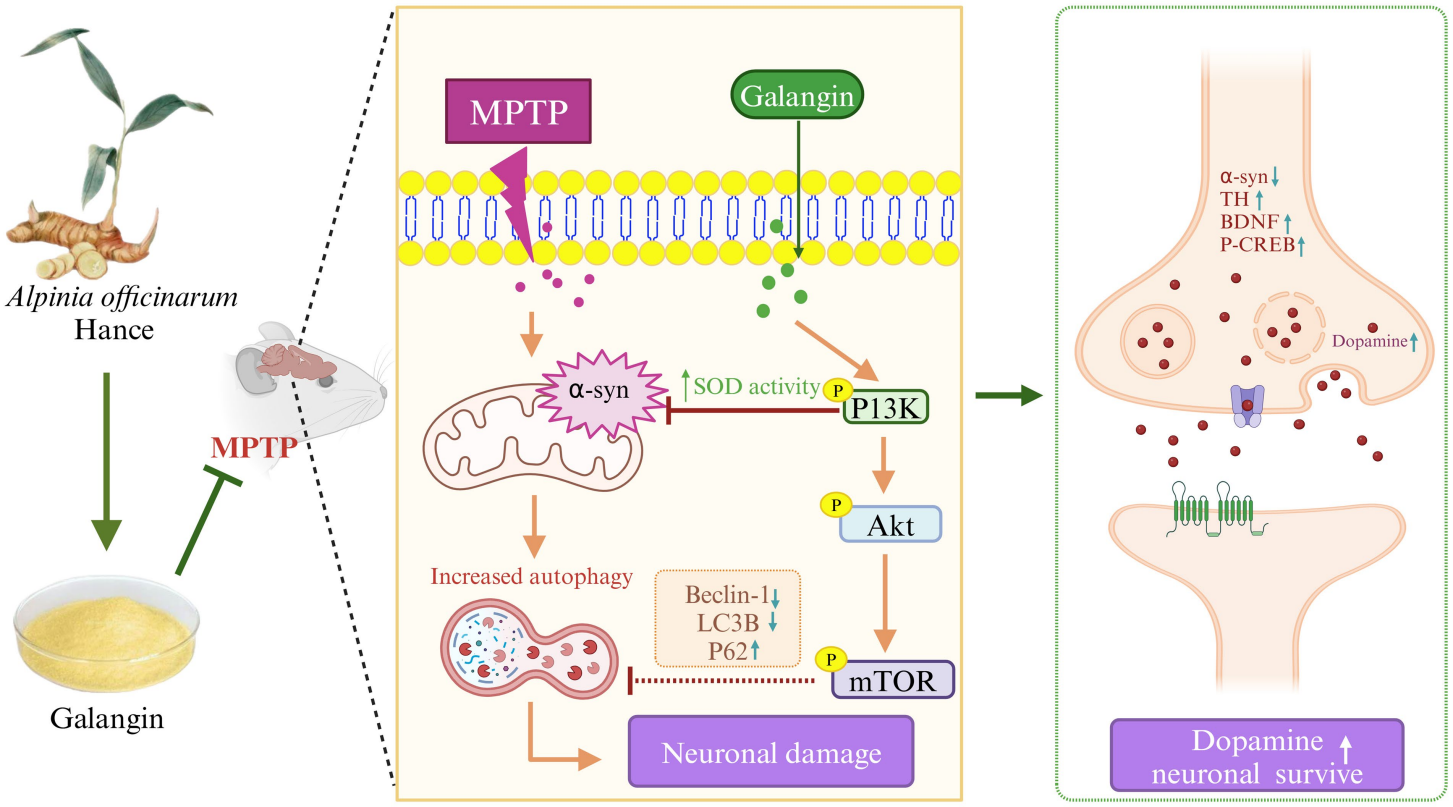
Galangin attenuated MPTP-induced brain tissue injury in PD model mice, and the related mechanism may be related to the PI3K/AKT signaling pathway. MPTP-induced neuronal injury in the brain of PD model mice exhibited α-syn aggregation, decreased TH expression, increased inflammatory factor levels, and high autophagy levels. Activation of PI3K/AKT signaling pathway could alleviate the autophagy level to a certain extent, reduce inflammation, decrease α-syn aggregation and promote TH expression, and finally repair the activity of dopaminergic neurons. Therefore, we demonstrate the preventive and therapeutic potential of Galangin in attenuating PD, at least in part, by regulating the PI3K/AKT signaling pathway. MPTP, N-methyl-4-phenyl-1,2,3,6-tetrahydropyridine; PD, Parkinson’s disease; α-syn, α-synuclein; TH, tyrosine hydroxilase; PI3K, Phosphatidylinositol 3-Kinase; AKT, Protein Kinase B; mTOR, mechanistic target of rapamycin.

Previous studies have shown that abnormal autophagy function is strongly associated with a variety of diseases, including neurodegenerative diseases, tumors, and other diseases (Kiriyama and Nochi, 2015). During MPTP-induced PD events, a substantial accumulation of *α*-syn and dysfunctional organelles occurs, causing the activation of autophagy. Studies suggest that autophagy is involved in the entire process of PD pathogenesis (Dehay et al., 2010). Autopsies of patients with PD revealed an accumulation of autophagosomes accompanied by the absence of lysosomal markers in dopaminergic neurons (Heras-Sandoval et al., 2014). The PI3K/AKT/mTOR pathway may be affected to varying degrees in the brains of PD patients, and autopsy results of PD patients have revealed decreased activity of phosphorylated AKT in dopaminergic neurons; however, some studies have suggested that autophagy in the course of PD may be independent of the PI3K/AKT/mTOR pathway leading to neuronal damage (Mancuso and Navarro, 2015), and further research is needed to determine whether autophagy is involved in the pathogenesis of PD by affecting the PI3K/AKT/mTOR pathway. However, both network analysis and transcriptomic results suggest that Galangin treatment of PD mice is closely related to the PI3K/AKT pathway. In order to verify this conclusion, we established a mouse model of PD by MPTP, and evaluated the anti-PD effect of Galangin by roller test, TH, α-syn, autophagy and other indicators. Results demonstrated that Galangin improved motor coordination in PD mice and that the neuronal morphology and organization in the hippocampus and striatum were significantly better in the treatment group compared to the model group. Notably, the number of nistids and neurotrophic factor levels of BDNF and CREB were elevated, suggesting that Galangin can alleviate the neuronal damage caused by MPTP. The *α*-syn protein S129D mutant increases TH phosphorylation and DA synthesis (Hua et al., 2015; Xu et al., 2015). TH provides instructions for synthesizing DA in dopaminergic neurons, which is essential for the normal functioning of the nervous system. In the PD model, it was found that the accumulation or loss of *α*-syn would lead to dysregulation of TH activity in the brain (Farrell et al., 2014). Our previous experimental studies also found that after the use of autophagy inhibitors to inhibit autophagy activity, TH activity was increased, while α-syn expression was reduced, and the neuronal damage was attenuated (Zhang et al., 2016). Notably, Beclin-1, LC3B and P62 are widely employed as autophagy biomarkers. LC3B is the first autophagosome membrane protein found in eukaryotic cells. It is an important component of autophagosome, and its content is positively correlated with the number of autophagosomes (Chmid et al., 2013). Beclin-1 is a promoter of autophagy, and its activity is regulated by phosphorylation and ubiquitination in various ways, thus regulating autophagy level (Park et al., 2018; Hill et al., 2019). Autophagy receptor P62 recognizes ubiquitin-labeled proteins and organelles that degrade autophagy receptors and interacting adaptors during selective autophagy (Chmid et al., 2013). Dysautophagy can lead to accumulation of abnormal proteins or organelles in cells, causing PD. Moderate activation of autophagy can be used for the treatment of PD, while excessive autophagy can cause neuronal death. However, how to regulate autophagy for PD treatment is still a research difficulty. Herein, we observed that DA and its metaboliets DOPAC and HVA levels, TH and P62, P-PI3K, P-mTOR and P-AKT expression were decreased, and а-syn, Beclin-1, LC3B were significantly increased in the PD mice compared with the control mice. In contrast, treatment with Galangin abrogated the PD-mediated increase in autophagy levels, at the same time, а-syn decreased, DA and its metaboliets DOPAC and HVA levels, TH, P-PI3K, P-mTOR and P-AKT expression evaluated. These results indicated that Galangin may alleviated the autophagy and improved the motor dysfunction PD mice (Figure 10).

Neuroinflammation is considered to be an important factor in the pathogenesis of PD (Lee et al., 2019; Earls and Lee, 2020). In many animal models of PD, peripheral inflammation has been shown to exacerbate the degeneration of dopaminergic neurons. Oxidative stress, mitochondrial dysfunction, abnormal aggregation of *α*-syn, and synergistic effects of endogenous neurotoxins can also exacerbate chronic inflammation and neuronal death (Sun et al., 2019; Liddelow et al., 2017). Research indicates that when the NLRP3 inflammasome detects α-syn aggregation, it activates caspase-1, which subsequently induces the release of pro-inflammatory cytokines such as IL-1β and IL-18. This process intensifies the inflammatory response in PD and promotes further injury to dopaminergic neurons and excessive α-syn aggregation. Knockout the inflammasome can make MPTP-induced PD mouse models characteristic of loss of anti-nigra dopaminergic neurons associated with decreased secretion of IL-1β and IL-18 (Yan et al., 2015; Teleanu et al., 2022). Therefore, antioxidant have become an important idea in PD treatment, which helps to protect neurons and delay neurodegeneration, so as to delay the development of the disease. Catalase (CAT) and GSH-Px have antioxidant activities, and the decrease of CAT and GSH-Px levels in PD patients is an important reason for the mass production of free radicals, which accelerates the damage of dopaminergic neurons and accelerates the development of the disease (Percario et al., 2020; Leathen et al., 2022). Our experimental results indicate that the PD model group exhibited significant oxidative stress damage. Specifically, compared to the control group, the levels of GSH-Px mRNA, GSH-Px and SOD significantly decreased, while the level of MDA significantly increased. Compared to the PD model group, the different doses of Galangin treatment groups significantly upregulated the levels of GSH-Px mRNA, GSH-Px and SOD, and downregulated the level of MDA. These results further suggest that Galangin has a protective effect on MPTP-induced PD model mice, which may be related to its antioxidant properties. Furthermore, our results also found that the protein or mRNA levels of IL-1β, IL-6 and TNF-α were elevated in PD model mice. Galangin administration partially restored the levels of IL-1β, IL-6 and TNF-α. We suggest that PD mice suffering from MPTP toxicity have a redox imbalance in the brain. The abnormal accumulation of IL-1β, IL-6 and TNF-α disrupted the normal structure of the brain and cognitive function.

To further explore the molecular mechanism of Galangin intervention in PD, we conducted transcriptomic analysis of differentially expressed genes in the midbrain tissues of mice from both the high-dose Galangin-treated group and the model group. Following this, we performed GO analysis and enrichment analysis of differentially expressed genes. The GO analysis results of this study showed that compared with the model group, the functions of the differential genes in the Galangin treated group were mainly focused on cell cycle, DNA replication, cytoplasm, and identical protein binding. In addition, the enrichment analysis results of KEGG signal pathway showed that compared with the model group, the differential genes involved in the Galangin treatment group were mainly PI3K/AKT signaling pathway, MAPK signaling pathway, TNF signaling pathway and cellular senescence. These results suggest that the anti-PD effect of Galangin may be related to the regulation of cell function and inflammatory response. Furthermore, the results of differential gene analysis in this study showed that after the intervention of Galangin in PD, the top 10 genes up-regulated in the midbrain of PD mice involving Gsta3, LOC115487393, Pik3r1, Gm40703, Rgs1, Gm52312, Gm46784, Cd200r4, Gprc5c, Gm30657. Conversely, down-regulated genes included Cpa4, Tnf, Cdc6, Pbk, Lrrl, Esco2, Nfkb1, Tlr9, Ankle1 and Pclaf. Most of these genes are related to cell growth and apoptosis, cancer, inflammation and so on. Among them, Nfkb1 is a widespread and important transcription factor involved in T cell activation, gene expression involved in a variety of biological functions such as immune regulation and cell adhesion, and is associated with a variety of immune diseases (Camblor et al., 2022; Dange et al., 2015; Ladygina et al., 2013; Jiang et al., 2022). According to the RT-PCR test, compared with the PD model group, Cpa4, Lrr1, Tlr9, spc24, Nfkb1, Tnf, Gsta3, Pik3r1 and Esco2 in Galangin group were significantly down-regulated, while Gpc5c was up-regulated. The results were consistent with those of transcriptomic analysis. Therefore, we suggest that Galangin can improve motor coordination of PD mice is closely related to activate the PI3K/AKT pathway and anti-inflammation.

Our concentration analysis of the KEGG pathway in network analysis indicates that the PI3K/AKT pathway is one of the primary anti-PD pathways influenced by Galangin, along with cellular senescence, the MAPK signaling pathway, the TNF signaling pathway, and the p53 signaling pathway. However, we only studied PI3K/AKT and did not conduct relevant studies on other pathways. Additionally, the other difference-related genes do not appear in the PI3K/AKT pathway, and the correlation with PD is not yet clear, so it needs to be verified in the next step. There will be further explanation as to whether these signaling pathways are involved in PD. Furthermore, these pathways are complex, and more intensive studies can be conducted in the future to explore the specific mechanisms signaling Galangin exerts protective effects against PD.

## 5 Conclusion

In conclusion, this study confirmed that Galangin treatment promoted motor coordination and attenuated the damage of dopaminergic neurons in PD model mice. The therapeutic effect of H-Galangin was more significant than that of L-Galangin and M-Galangin. Additional exploration of the mechanism of the protective effect of Galangin on the PD model mice through network analysis, molecular docking and transcriptomic analysis suggested that it may involve the activation of the PI3K/AKT signaling pathway. Moreover, Galangin inhibited autophagy-related protein levels and promoted neurotrophic factor levels of BDNF and CREB. These results suggest that Galangin could be developed as a drug for PD therapy.

## Data Availability

The original contributions presented in the study are included in the article/Supplementary material, further inquiries can be directed to the corresponding authors.

## Footnotes

^1^ https://old.tcmsp-e.com/tcmspsearch.php

^2^ http://www.swisstargetprediction.ch/

^3^ http://www.Lilab-ecust.cn/pharmmapper/

^4^ https://www.uniprot.org/

^5^ https://www.genecards.org/

^6^ https://www.disgenet.org/

^7^ https://www.omim.org/

^8^ https://bioinfogp.cnb.csic.es/tools/venny/

^9^ https://string-db.org/cgi/input.pl

^10^ https://david.ncifcrf.gov/

^11^ http://www.bioinformatics.com.cn/
